# Comparison of Ticagrelor and Clopidogrel in Elective Coronary Stenting: A Double Blind Randomized Clinical Trial

**DOI:** 10.1155/2023/5544440

**Published:** 2023-12-26

**Authors:** Mohammadjavad Mehdizadeh Parizi, Reza Golchin Vafa, Amin Ahmadi, Reza Heydarzade, Mehrdad Sadeghi, Amin Khademolhossseini, Farhang Amiri, Soroush Khoshnood Mansorkhani, Ali Tavan, Nazanin Hosseini, Mohammad Montaseri, Seyed Ali Hosseini, Javad Kojuri

**Affiliations:** ^1^Professor Kojuri Cardiology Clinic, Shiraz, Iran; ^2^Cardiology Department, Shiraz University of Medical Sciences, Shiraz, Iran; ^3^Clinical Education Research Center, Shiraz University of Medical Sciences, Shiraz, Iran

## Abstract

**Background:**

Dual antiplatelet therapy with a P2Y12 inhibitor (e.g., clopidogrel and ticagrelor) and aspirin is recommended for at least one year after percutaneous coronary intervention (PCI) to prevent further myocardial infarction and stent thrombosis as the major adverse effects of PCI.

**Methods:**

This randomized clinical trial was conducted from October 2022 to March 2023. Patients who had undergone elective PCI were included in the study. Patients were randomized into two different groups. One group took ASA 80 mg and clopidogrel 75 mg once daily, while the other took ASA 80 mg once daily and ticagrelor 90 mg twice daily. After six months of close follow-up, patients were asked to score their dyspnea on a 10-point Likert scale. They were also asked about dyspnea on exertion, paroxysmal nocturnal dyspnea (PND), bleeding, and the occurrence of major adverse cardiovascular events (MACEs).

**Results:**

223 patients were allocated to the clopidogrel group and 214 to the ticagrelor group. In the ticagrelor group, 95 patients (44.3%) reported dyspnea at rest, compared with only 44 patients (19.7%) in the clopidogrel group (*P* < 0.001). MACEs occurred in 7 patients (2.8%) in the ticagrelor group, compared with 16 (7.6%) in the clopidogrel group (*P* = 0.031). Eight patients (3.8%) reported bleeding with ticagrelor, as did seven (3.2%) with clopidogrel (*P* = 0.799).

**Conclusions:**

New-onset dyspnea was recorded more frequently with ticagrelor than clopidogrel, yet fewer MACEs occurred with ticagrelor (ClinicalTrials.gov number: NCT05858918).

## 1. Introduction

Coronary artery disease (CAD) is one of the most common causes of death worldwide [1]. CAD has a wide range of clinical presentations, including asymptomatic atherosclerosis, stable angina, unstable angina, non-ST-elevation myocardial infarction (NSTEMI), and ST-elevation myocardial infarction (STEMI) [2]. Two invasive strategies are available for revascularization in patients with CAD: percutaneous coronary intervention (PCI) and coronary artery bypass grafting (CABG) [3]. Lately, there has been a dramatic increase in the application of PCI as a treatment for CAD [4]. Recent studies have shown an improvement in the quality of life of patients with CAD who have undergone PCI [5, 6]; however, several studies showed that the post-CABG mortality rate is lower than the mortality rate after PCI in patients with multivessel disease. Nevertheless, PCI is the treatment of choice for patients with single-vessel CAD [7].

Dual antiplatelet therapy (DAPT) with a P2Y12 inhibitor (e.g., clopidogrel, prasugrel, and ticagrelor) and aspirin are considered the cornerstone of secondary prevention in patients with acute coronary syndromes (ACS) with or without STEMI and patients with stable coronary artery disease undergoing PCI [8]. DAPT is recommended for at least 6 months in patients who underwent elective PCI due to stable coronary artery disease to effectively prevent subsequent myocardial infarction and stent thrombosis, one of the major adverse effects of PCI [9]. It is notable to mention that aspirin should be continued lifelong [10, 11]. Various P2Y12 inhibitors are available, and selecting a specific DAPT regimen is challenging.

Clopidogrel is a prodrug that requires hepatic conversion to be activated, which leads to a delay in the onset of action. The effectiveness of platelet inhibition by clopidogrel varies among individuals, and more than one-third of the patients are considered “clopidogrel nonresponders” who achieve minimal platelet inhibition when using this medication [12–14]. The risks of bleeding and stent thrombosis are the known disadvantages of clopidogrel use in post-PCI patients [15–17].

Ticagrelor, a reversible P2Y12 receptor antagonist, does not require hepatic activation. However, the PLATelet inhibition and patient outcomes (PLATO) trial showed that ticagrelor has significant benefits compared to clopidogrel in reducing total death, averting stent thrombosis, and preventing cardiovascular events [18, 19]. Others believe that, despite its greater antiplatelet potency, ticagrelor does not reduce the rate of major adverse cardiovascular events (MACE) much more than clopidogrel, especially in patients with chronic coronary syndrome, in whom the recommended antiplatelet is still clopidogrel [19, 20]. Although Li and colleagues showed no significant differences between ticagrelor and clopidogrel in provoking major bleeding [21], others concluded that ticagrelor is associated with a significantly higher rate of minor and major bleeding and dyspnea when compared to clopidogrel [22]. Hence, controversy persists in the literature regarding the difference in bleeding risk between clopidogrel and ticagrelor [23].

Due to the mentioned controversies, this article aimed to compare two P2Y12 inhibitors, clopidogrel and ticagrelor, regarding their efficacy and side effects among patients who underwent PCI.

## 2. Methods

We gathered clinical data for this Randomized Clinical Trial (RCT) study from patients referring to Professor Kojuri's Cardiovascular Clinic (Shiraz, Iran, e-mail: kojurij@yahoo.com, web page: https://kojuriclinic.com) from October 2022 to March 2023. The inclusion criterion was undergoing elective percutaneous coronary intervention (PCI) based on the 2021 ACC/AHA/SCAI Guideline for Coronary Artery Revascularization [24] (Table 1). Patients with hypersensitivity to ticagrelor, clopidogrel, or other contraindications, such as active pathological bleeding, were excluded from the study, as patients who used anticoagulants during their medical therapy.

Basic clinical information of all patients and comorbidities such as diabetes mellitus (diagnosed based on ADA 2018 criteria [25]), hypertension (defined as systolic blood pressure >130 mmHg or diastolic blood pressure >80 mmHg based on 2017 ACC/AHA hypertension guidelines [26]), dyslipidemia, anemia (defined according to 1968 WHO definition [27]), asthma (diagnosed based on 2007 NAEPP criteria [28]), chronic obstructive pulmonary disease (diagnosed according to 2020 GOLD criteria [29]), and atrial fibrillation (AF) were recorded. We also noted other clinical data such as body mass index (normal: 18.5–24.9; overweight: 25–29.9; and obesity: higher than 30 kg/m^2^), left ventricle ejection fracture (LVEF), heart failure status (classified based on the 2001 ACC/AHA heart failure classification [30]), history of using tobacco within the past three months, and past drug history. We defined major bleeding according to the Thrombolysis in Myocardial Infarction (TIMI) criteria: intracranial bleeding, hemorrhage with a hemoglobin decrease of at least 5 g/dL, or fatal bleeding that caused death within seven days [31].

Patients scored their dyspnea on a 10-point Likert scale before undergoing PCI. Scores 1–3 were mild, 4–6 were moderate, and 7–10 were severe. We randomized patients using the block randomization method with a block size = 4 into two groups. One group received acetylsalicylic acid (ASA) 80 mg daily and clopidogrel 75 mg daily (Sanofi Co.; brand name: Plavix) and the other received ASA 80 mg daily and ticagrelor 90 mg twice daily (Abidi Co.; brand name: Brilavus).

We thoroughly explained the study objectives and protocols to the patients and obtained informed consent, excluding those who did not wish to participate. We asked the participants to take their medications regularly and call us immediately if they faced any medical problems for further investigation. To check drug compliance and complications, we followed up on the patients via monthly phone calls and in-person visits after one, three, and six months of starting medications.

After six months of close follow-up, patients scored their post-PCI dyspnea on the 10-point Likert scale. We also asked them about dyspnea on exertion, paroxysmal nocturnal dyspnea (PND), bleeding (e.g., gastrointestinal bleeding), and MACEs, defined as an acute coronary syndrome (ACS), stroke, revascularization, hospitalization due to heart failure, or cardiac death [29]. The primary endpoints of the study are MACE, dyspnea at rest, dyspnea on exertion, orthopnea, and PND. Adherence and major bleeding are secondary endpoints in this study.

For statistical analysis, we used IBM SPSS software version 25. To analyze the study data, we used the intention-to-treat method. As a result, patients were included in the analysis based on the originally assigned group, regardless of the received medication. We summarized data with a normal distribution using the mean ± standard deviation. We also reported the variables' odds ratio (OR) and 95% confidence interval (CI). Independent-sample *t*-tests and one-way ANOVA were used to analyze parametric data, while the Mann–Whitney U and Kruskal–Wallis tests were used for nonparametric data. Values of *P* < 0.05 were considered significant.

The Ethics Committee of Shiraz University of Medical Sciences approved the study protocol (IR.SUMS.MED.REC.1401.351).

## 3. Results

**Figure 1:**
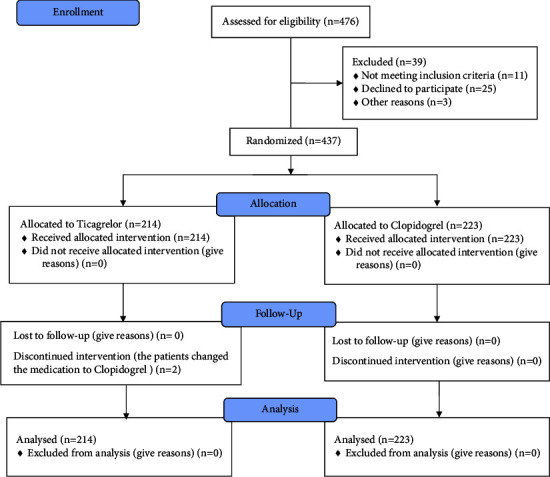
CONSORT flowchart of the study.

Four hundred seventy-six eligible patients who visited our cardiovascular clinic were selected initially. Thirty-nine patients were excluded according to the mentioned exclusion criteria. Two patients changed from ticagrelor to clopidogrel due to the onset of dyspnea. No patients were hospitalized due to dyspnea. The remaining 437 patients (154 women and 283 men) were included in this study, 233 were allocated to the “clopidogrel group” and 214 to the “ticagrelor group” (Figure 1).

The mean age of the patients was 62 ± 9 years. The groups' baseline characteristics are compared in Table 2, and the baseline medications are compared in Table 3.

**Figure 2:**
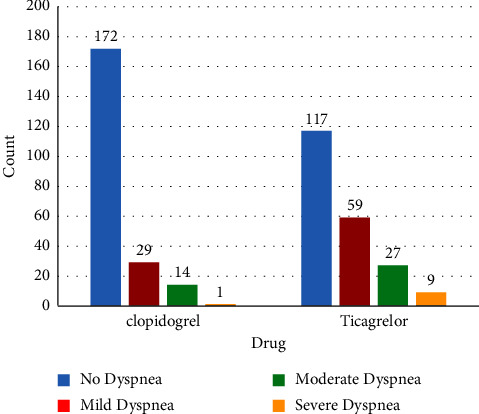
Prevalence of dyspnea in the ticagrelor and clopidogrel groups.

In the ticagrelor group, 95 patients (44.8%) reported dyspnea at rest, compared with 44 patients (19.7%) in the clopidogrel group (OR = 3.3, 95% CI = 2.50–5.06, and *P* < 0.001). In the ticagrelor group, 40% of patients with dyspnea after PCI had no dyspnea before PCI. In other words, 40% of patients in the ticagrelor group had new-onset dyspnea after PCI, compared with 13.5% of patients in the clopidogrel group (OR = 7.89, 95% CI = 3.26–19.11, and *P* = 0.002). In the ticagrelor group, 9 (4.2%) patients reported severe dyspnea, 27 (12.6%) reported moderate dyspnea, and 59 (27.5%) reported mild dyspnea. In contrast, in the clopidogrel group, only one patient (0.4%) complained about severe dyspnea, 14 (6.2%) reported moderate dyspnea, and 29 (13%) reported mild dyspnea (Figure 2).

Orthopnea was experienced by 22 patients (10.2%) in the ticagrelor group and 13 patients (5.8%) in the clopidogrel group (OR = 1.81, 95% CI = 0.89–3.7, and *P* = 0.113). Thirteen patients (6%) in the ticagrelor group had PND, as did six patients (2.6%) in the clopidogrel group (OR = 2.26, 95% CI = 0.84–6.07, and *P* = 0.106) (Table 4).

A higher percentage of patients in the clopidogrel group (91.9%) adhered completely to their medication compared to the ticagrelor group (87.8%). However, this difference was not statistically significant (*P* = 0.271). Among the patients of the ticagrelor group, 12 (5.6%) continued to use their medications with minor interventions such as caffeine or herbal medications. Six patients (2.8%) in this group changed their medicines due to dyspnea or stopped ticagrelor completely. In the clopidogrel group, 11 patients (4.9%) maintained their medications with slight interventions. Only one (0.4%) switched to another P2Y12 inhibitor, and none stopped taking their medications (Table 5).

A MACE was seen in six patients of the ticagrelor group (2.8%), all presenting with ACS. On the other hand, there were 16 events (7.6%) in the clopidogrel group, consisting of 14 cases of ACS (6.2%), one heart failure (0.4%), one cerebrovascular event (0.4%), and one cardiac death (0.4%). The difference was statistically significant (OR = 2.86, 95% CI = 1.1–7.4, and *P* = 0.031). In the ticagrelor group, eight patients (3.8%) reported minor bleeding, as did seven (3.2%) in the other group (*P* = 0.799). There was no significant difference between the groups regarding noncardiac deaths (Table 6).

## 4. Discussion

This randomized clinical trial compared clopidogrel and ticagrelor, two P2Y12 inhibitors, regarding their efficacy and side effects among patients who underwent elective PCI, with a follow-up period of six months. Our study showed that new-onset dyspnea was significantly more common among patients treated with ticagrelor rather than clopidogrel, though fewer MACEs occurred in the ticagrelor group.

Storey et al. found that dyspnea among patients consuming ticagrelor was significantly more common than those using clopidogrel [32]. While our study demonstrated that many ticagrelor and clopidogrel users had dyspnea at rest, the percentage of patients who developed new-onset dyspnea after PCI was significantly higher in the ticagrelor group. In another study, Iqbal Wani et al. found that the prevalence of orthopnea and PND in clopidogrel-consuming patients was insignificantly higher than in those using ticagrelor [33]. Conversely, our data analysis showed that the prevalence of orthopnea and PND was higher in the ticagrelor group; however, the difference was insignificant.

When considering medication adherence, which means how actively patients are consuming their prescribed medications [34], it was reported by Bergmeijer et al. that nearly one-quarter of patients taking ticagrelor as a part of their treatment for ACS discontinued ticagrelor or changed it to other types of medications within one year of consumption, mostly due to dyspnea or bleeding [35]. While we maintained medications in both groups with minor interventions, adherence was much better in the patients using clopidogrel than those using ticagrelor. Since clopidogrel can be used once daily, adherence seems to be achieved more easily than ticagrelor, which must be taken twice daily.

Our study showed that MACEs were less common in the group taking ticagrelor, suggesting that ticagrelor may reduce the risk of MACEs more efficiently than clopidogrel. Xue et al. found no remarkable differences in benefiting from ticagrelor or clopidogrel use in patients who underwent PCI due to ACS in multiple-vessel disease. However, patients with single-vessel disease treated with ticagrelor had less adverse outcomes (such as MACE, hospitalization, and bleeding) than those taking clopidogrel [36]. In another study comparing ticagrelor and clopidogrel in ACS patients, Wallentin et al. found that patients who used ticagrelor were less likely to experience vascular-related death, MI, or overall MACE, when compared to clopidogrel [37]. The meta-analysis and systemic review of Andreou et al. concluded that among the patients who were treated with dual antiplatelet therapy, the occurrence of MACE was not significantly different between ticagrelor and clopidogrel users; however, patients who were treated with triple therapy and had ticagrelor in their regimen experienced more MACEs than clopidogrel users [38]. In another study in this controversial field, Varenhorst et al. found that patients consuming ticagrelor had a significantly lower probability of sudden death when compared to clopidogrel, even though the risks of acute MI and heart failure were equal [39].

While we discovered no significant inequality between ticagrelor and clopidogrel regarding bleeding-related complications and noncardiac death, Misumida et al., in their meta-analysis and systemic review, reported that the risk of major bleeding among eastern Asian patients with previous ACS consuming ticagrelor was higher in comparison to the ones taking clopidogrel (OR: 1.52). They also reported that this difference could be attributed to the higher prevalence of renal failure and low BMI in the Asian population [40]. Becker et al. concluded that the total risk of major bleeding in ACS patients consuming ticagrelor was similar to those using clopidogrel, but the risk of bleeding was not related to CABG or procedures. For instance, intracranial bleeding within the first thirty days in patients consuming ticagrelor was higher. According to that study, the risk of fatal bleeding was low, and there were no significant differences between the two groups regarding this type of bleeding [41]. In contrast to our findings, the meta-analysis and systemic review of Andreou et al. found that consuming ticagrelor as a part of either dual therapy or triple therapy contributed to a higher probability of bleeding complications than clopidogrel [38]. Finally, Varenhorst et al. found no remarkable differences regarding nonvascular death between ticagrelor and clopidogrel users [39].

### 4.1. Study Limitations

One of the limitations of our study was that we followed the patients for only six months after starting the medications. Future studies with longer follow-up periods can reveal more information about the advantages and disadvantages of these medications. Attendance of the patients at the clinic can increase the accuracy of data gathering. Multicenter clinical trials are needed to minimize the effects of confounding factors and provide more reliable data.

## 5. Conclusions

This study showed that the prevalence of dyspnea among patients treated with ticagrelor was higher than that of those using clopidogrel. However, most of the patients in both groups experienced mild dyspnea. Interestingly, this study demonstrated that ticagrelor can induce more new-onset dyspnea in patients than clopidogrel. Although the patients who used clopidogrel had better medication adherence than the ones taking ticagrelor, the prevalence of MACE was less with ticagrelor. There were no significant differences between clopidogrel and ticagrelor regarding the bleeding risk.

## Figures and Tables

**Table 1:** Indications of PCI in chronic CAD.

(1) Symptoms of angina refractory to medical therapy
(2) LT main stenosis in patients with syntax score ≤ 33
(3) LT main stenosis in patients with syntax score > 33 who are not suitable CABG candidate
(4) Multivessels coronary artery disease with lvef > 50%
(5) Multivessels coronary artery disease with lvef ≤ 50% who are not suitable CABG candidate

**Table 2:** Baseline characteristics of the study participants, mean ± SD or *n* (%).

	Clopidogrel (*n* = 223)	Ticagrelor (*n* = 214)	*P* value
Age, years	63 ± 10	62 ± 9	0.502
Body mass index (kg/m^2^)	27.2 ± 4.1	27.3 ± 4.3	0.727
Male, *n* (%)	153 (68.6%)	130 (60.7%)	0.085
Hypertension, *n* (%)	141 (63.2%)	122 (57%)	0.184
Diabetes mellitus, *n* (%)	75 (33.6%)	64 (29.9%)	0.403
Smoker, *n* (%)	51 (22.9%)	56 (26.2%)	0.423
Dyslipidemia, *n* (%)	83 (37.2%)	107 (50%)	0.007
COPD, *n* (%)	9 (4%)	16 (7.5%)	0.150
Asthma, *n* (%)	5 (2.2%)	12 (5.6%)	0.085
HFrEF, *n* (%)	21 (9.4%)	7 (3.3%)	0.008
Atrial fibrillation, *n* (%)	4 (1.8%)	0 (0%)	0.124
Anemia, *n* (%)	7 (3.1%)	14 (6.5%)	0.118

COPD: chronic obstructive pulmonary disease; HFrEF: heart failure with reduced ejection fraction.

**Table 3:** Baseline medications of patients in the clopidogrel and ticagrelor groups, *n* (%).

	Clopidogrel	Ticagrelor	*P* value
	(*n* = 223)	(*n* = 214)	
Statins	219 (98.2%)	210 (98.1%)	1
Beta-blockers	154 (69.1%)	132 (61.7%)	0.109
Diuretics	29 (13%)	22 (10.3%)	0.456
Calcium channel blockers	47 (21.1%)	41 (19.2%)	0.635
Angiotensin-converting enzyme inhibitors	32 (14.3%)	13 (6.1%)	0.005
Angiotensin II receptor blockers	86 (38.6%)	93 (43.5%)	0.331
Nitrates	75 (33.6%)	106 (49.5%)	0.001
Proton pump inhibitors	78 (35%)	85 (39.7%)	0.323

**Table 4:** Symptoms of patients in the clopidogrel and ticagrelor groups.

	Clopidogrel (*n* = 223)	Ticagrelor (*n* = 214)	*P* value
Dyspnea at rest, *n* (%)	44 (19.7%)	95 (44.3%)	<0.001
Mild dyspnea, *n* (%)	29 (13%)	59 (27.5%)	
Moderate dyspnea, *n* (%)	14 (6.2%)	27 (12.6%)	
Severe dyspnea, *n* (%)	1 (0.4%)	9 (4.2%)	
Dyspnea on exertion, *n* (%)	49 (21.9%)	53 (24.7%)	0.572
Orthopnea, *n* (%)	13 (5.8%)	22 (10.2%)	0.113
Paroxysmal nocturnal dyspnea, *n* (%)	6 (2.6%)	13 (6%)	0.106

**Table 5:** Interventions for symptoms in the clopidogrel and ticagrelor groups.

Intervention	Clopidogrel	Ticagrelor	*P* value
	(*n* = 223)	(*n* = 214)	
No intervention	205 (91.9%)	188 (87.8%)	0.146
Minor interventions (e.g., caffeine usage)	11 (4.9%)	12 (5.6%)	0.061
Changed medication	1 (0.4%)	6 (2.8%)	0.056
Medication stopped by the patient	0	6 (2.8%)	0.388
Missing	6 (2.6%)	2 (0.9%)	0.069

**Table 6:** Outcomes of patients in the clopidogrel and ticagrelor groups.

	Clopidogrel (*n* = 223)	Ticagrelor (*n* = 214)	*P* value
Major adverse cardiac events	17 (7.6%)	6 (2.8%)	0.031
Acute coronary syndrome, *n* (%)	14 (6.2%)	6 (2.8%)	
Heart failure, *n* (%)	1 (0.4%)	0	
Cerebrovascular events, *n* (%)	1 (0.4%)	0	
Cardiac death, *n* (%)	1 (0.4%)	0	
Noncardiac death, *n* (%)	3 (1.3%)	1 (0.4%)	0.371
Bleeding, *n* (%)	7 (3.2%)	8 (3.8%)	0.799

## Data Availability

Data are available in professor Kojuri cardiology clinic registry and will be available upon reasonable request.
